# Implications of trimethylamine N-oxide (TMAO) and Betaine in Human Health: Beyond Being Osmoprotective Compounds

**DOI:** 10.3389/fmolb.2022.964624

**Published:** 2022-08-26

**Authors:** Ashal Ilyas, Yasanandana Supunsiri Wijayasinghe, Ilyas Khan, Nourhan M. El Samaloty, Mohd Adnan, Tanveer Ali Dar, Nitesh Kumar Poddar, Laishram R. Singh, Hemlata Sharma, Shahanavaj Khan

**Affiliations:** ^1^ Department of Biotechnology, Invertis University, Bareilly, Uttar Pradesh, India; ^2^ Department of Biochemistry and Clinical Chemistry, Faculty of Medicine, University of Kelaniya, Ragama, Sri Lanka; ^3^ Department of Mathematics, College of Science Al-Zulfi, Majmaah University, Al-Majmaah, Saudi Arabia; ^4^ Department of Pharmacology, Toxicology and Biochemistry, Faculty of Pharmacy, Future University in Egypt, Cairo, Egypt; ^5^ Department of Biology, College of Science, University of Hail, Hail, Saudi Arabia; ^6^ Department of Clinical Biochemistry, University of Kashmir, Srinagar, Jammu and Kashmir, India; ^7^ Department of Biosciences, Manipal University Jaipur, Jaipur, Rajasthan, India; ^8^ Dr. B.R. Ambedkar Center for Biomedical Research, University of Delhi, Delhi, India; ^9^ Department of Pharmaceutics, College of Pharmacy, King Saud University, Riyadh, Saudi Arabia; ^10^ Department of Medical Lab Technology, Indian Institute of Health and Technology (IIHT), Saharanpur, Uttar Pradesh, India

**Keywords:** osmolytes, chemical chaperones, TMAO, betaine, choline, cardiovascular disease, liver disease

## Abstract

Osmolytes are naturally occurring small molecular weight organic molecules, which are accumulated in large amounts in all life forms to maintain the stability of cellular proteins and hence preserve their functions during adverse environmental conditions. Trimethylamine N-oxide (TMAO) and N,N,N-trimethylglycine (betaine) are methylamine osmolytes that have been extensively studied for their diverse roles in humans and have demonstrated opposing relations with human health. These osmolytes are obtained from food and synthesized endogenously using dietary constituents like choline and carnitine. Especially, gut microbiota plays a vital role in TMAO synthesis and contributes significantly to plasma TMAO levels. The elevated plasma TMAO has been reported to be correlated with the pathogenesis of numerous human diseases, including cardiovascular disease, heart failure, kidney diseases, metabolic syndrome, etc.; Hence, TMAO has been recognized as a novel biomarker for the detection/prediction of several human diseases. In contrast, betaine acts as a methyl donor in one-carbon metabolism, maintains cellular S-adenosylmethionine levels, and protects the cells from the harmful effects of increased plasma homocysteine. Betaine also demonstrates antioxidant and anti-inflammatory activities and has a promising therapeutic value in several human diseases, including homocystinuria and fatty liver disease. The present review examines the multifarious functions of TMAO and betaine with possible molecular mechanisms towards a better understanding of their emerging and diverging functions with probable implications in the prevention, diagnosis, and treatment of human diseases.

## 1 Introduction

Osmolytes are low-molecular-weight organic molecules occurring naturally in living organisms that help sustain cell volume and have a propounding role in stress tolerance. The organisms ranging from microbes to higher plants and animals accumulate osmolytes in high concentrations during stress conditions in order to protect their cellular components (Burg and Ferraris, 2008; Wijayasinghe et al., 2017). Osmolytes belong to several chemical classes; sugars (e.g., glucose, sucrose, and trehalose), polyols (e.g., glycerol, sorbitol, mannitol, inositol), amino acids (e.g., glycine, arginine, proline, taurine), methylamines (e.g., trimethylamine N-oxide, glycine betaine), and urea (Yancey, 2005; Rabbani and Choi, 2018).

Osmolytes except urea function in maintaining the native folded conformation of proteins by increasing the near free energy of unfolded state of the protein (Khan et al., 2010). These small organic solutes are known to alter the cellular environment by interfering with the solvent properties (Gekko and Timasheff, 1981). Many osmolytes do not directly interact with proteins but exhibit osmoprotectant activity by excluding them from the protein surface (i.e., osmophobic effect), allowing proteins to fold correctly (Courtenay et al., 2000; Bruzdziak et al., 2013). In addition, osmolytes also function as chemical chaperones that assist the proper folding of vulnerable proteins (Diamant et al., 2001; Singh et al., 2007). Moreover, the osmolytes except urea are considered compatible solutes. They have two important attributes, which allow them to be chosen as protecting osmolytes during evolution: 1) they provide high stability for proteins against denaturation, and 2) they can accumulate at high concentrations in the cell without impeding the cellular functions (Singh et al., 2011). In contrast, intracellular accumulation of inorganic NaCl results in altered cellular activity (Burg and Ferraris, 2008). Thus, nature has chosen organic osmolytes over inorganic salts as osmoprotectants.

Among natural osmolytes, glycine betaine (simply betaine) and trimethylamine N-oxide (TMAO) have attracted much attention recently due to their involvement in human health. In this study, we recapitulate the updated knowledge on the chemistry and biology of glycine betaine and TMAO with respect to human health and disease.

## 2 Trimethylamine-N-oxide and betaine as vital osmoprotectants

**FIGURE 1:**
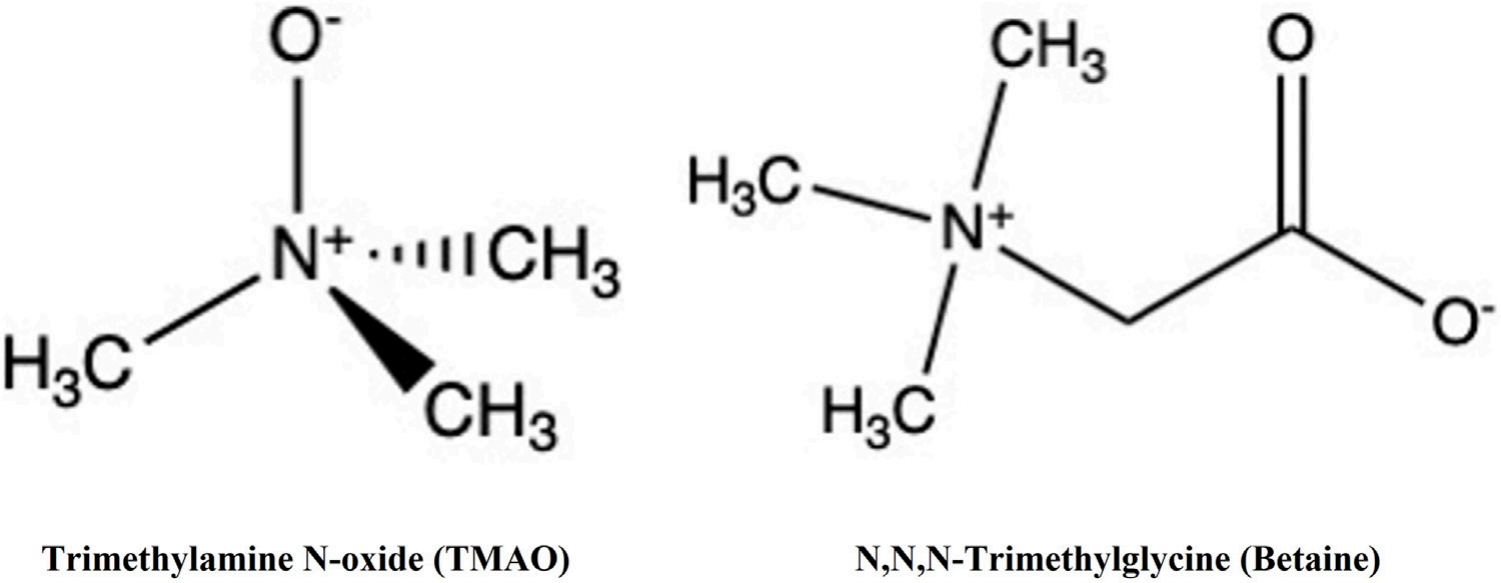
Structure of TMAO and betaine. Both TMAO and betaine belong to methylamine osmolytes.

TMAO and glycine betaine (N,N,N-trimethylglycine) (Figure 1) are widespread in nature. These osmolytes belong to the methylamine chemical group but possess distinct biochemical characteristics.

TMAO is abundant in many shallow and deep-sea fish (e.g., elasmobranchs, gadiform teleosts, etc.), which helps shield their cellular components from low temperature, high salinity, and high hydrostatic pressure in the surrounding environment (Raymond, 1994; Gillett et al., 1997; Treberg and Driedzic, 2002; Yancey et al., 2014). In particular, TMAO is accumulated in one half the concentration of urea in shallow-sea elasmobranchs to counteract the deleterious effects of urea on cellular proteins (Seibel and Walsh, 2002). Urea is not found in other marine organisms to any significant extent and is lower in deep-sea creatures compared to shallow living animals. However, TMAO is concentrated in deep-sea animals to counteract the effects of high hydrostatic pressure on cellular proteins.

On the other hand, glycine betaine is found in all living forms, from microorganisms to plants and animals. The biotic stress, such as drought, radiation, high salinity, extreme temperature, etc., triggers the synthesis and accumulation of betaine in plant cells (Ashraf and Foolad, 2007; Annunziata et al., 2019). Betaine helps to maintain the osmotic integrity in halophytes under high salt concentrations. For example, Poaceae and Chenopodiaceae plants contain betaine as a major osmoprotectant that allows them to survive during stressful environmental conditions (Ashraf and Foolad, 2007; Chen and Murata, 2008; Annunziata et al., 2019). Betaine is also shown to enhance plant drought and salt tolerance by mitigating oxidative stress (Ashraf and Foolad, 2007; Hasanuzzaman et al., 2014).

### 2.1 Trimethylamine-N-oxide and betaine in humans

**FIGURE 2:**
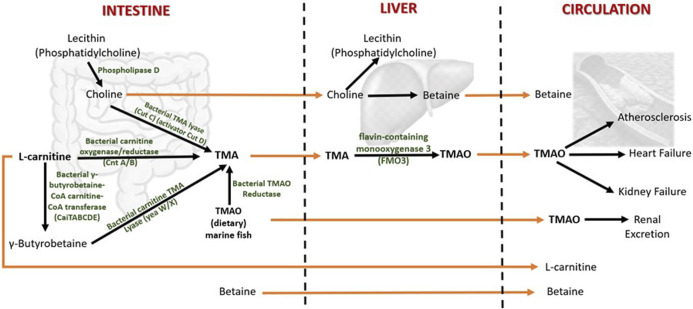
Metabolism of TMAO and betaine in humans. Both TMAO and betaine are procured from the diet and synthesized endogenously from the dietary constituents. The gut microbiota plays a central role in TMAO biosynthesis and thereby contributes significantly to plasma TMAO levels. TMA (trimethylamine) is an intermediate of TMAO metabolism. TMAO is eliminated from the body by excreting in the urine. The increased levels of circulating TMAO are found to be associated with several human diseases. The dietary choline is used to synthesize phosphatidylcholine (a phospholipid abundant in cell membranes), acetylcholine (a neurotransmitter), and betaine (a methyl donor). Betaine is eliminated by metabolism, and urinary excretion is insignificant.

In humans, TMAO is mostly biosynthesized from the unabsorbed dietary constituents containing trimethylamine moiety such as choline (concentrated in egg yolk and liver) and L-carnitine (predominantly in red meat) through the activity of inhabitant bacteria of the human large intestine (Li et al., 2018a) (Figure 2). Choline in food exists as free choline and choline esters (primarily phosphatidylcholine or lecithin) (Zeisel and Da Costa, 2009). Hydrolysis of lecithin by microbial phospholipase D releases choline (Chittim et al., 2019). The betaine/choline/carnitine transporters (BCCT) facilitate the uptake of choline, L-carnitine, and betaine in bacteria (Ziegler et al., 2010; Kivenson and Giovannoni, 2020).

The gut microbes first metabolize choline and L-carnitine to volatile trimethylamine (TMA). TMA is synthesized from choline by choline TMA-lyase (CutC)/activator CutD, and from dietary L-carnitine by two processes: 1) One-step conversation of L-carnitine to TMA by carnitine oxygenase/reductase (CntA/B) and 2) by two-step pathway that involves γ-butyrobetaine (γBB). Synthesis of γBB from L-carnitine by unidentified enzyme(s) which is (are) possibly encoded by *caiTABCDE* (or *caiABCD*) gene operon (Koeth et al., 2019; Rajakovich et al., 2021). However, the rate of microbial γBB production was reported to be approximately 1,000-fold higher than the rate of TMA formation (Koeth et al., 2014). γBB is converted to TMA by carnitine TMA lyase (yeaW/X) (Koeth et al., 2014; Rath et al., 2017). Moreover, the dietary TMAO can be reduced to TMA by bacterial TMAO reductase (Hoyles et al., 2018). A considerable amount of TMAO in humans comes from the consumption of seafood (Gatarek and Kaluzna-Czaplinska, 2021). The bacterial conversion of TMAO to TMA is the reason for the typical fishy smell in decaying seafood. In addition, betaine can be converted to TMA by selenocysteine-containing glycine betaine reductase (GrdH) (Rajakovich et al., 2021). However, this reaction is found to perform an insignificant role in TMA production in the human gut (Kivenson and Giovannoni, 2020; Rath et al., 2020).

The TMA generated in the intestine is then transported *via* portal circulation to the liver, where TMA is rapidly oxidized to TMAO by the flavin-containing monooxygenase 3 (FMO3). FMO3 is one of five functional FMOs abundantly expressed in the adult human liver (Hisamuddin and Yang, 2007). A fraction of TMAO present in food (e.g., marine fish) is absorbed unchanged into the portal circulation, and the rest is metabolized to TMA in the large intestine by the action of bacterial TMAO reductase (TorA) (Cho et al., 2017; Krüger et al., 2017; Hoyles et al., 2018). The majority (>95%) of circulating TMAO is excreted by the kidney (Al-Waiz et al., 1987). Unlike other organisms, TMAO in humans does not undergo further metabolism; hence, it is suggested that TMAO is a waste product of choline metabolism (Ufnal et al., 2015). Genetic mutations that give rise to the deficiency of FMO3 cause a disorder called trimethylaminuria, in which TMA is excreted in body fluids such as urine, sweat, and breath, with the characteristic fishy odor. Recent studies have found that elevated circulating TMAO is associated with several human diseases that will be discussed in the following sections.

On the other hand, plant food, such as wheat bran, wheat germ, quinoa, beets, spinach, and marine invertebrates is a significant source of betaine in humans (Craig, 2004; Ross et al., 2014). Betaine is rapidly absorbed in the intestine (Schwahn et al., 2003). In addition, betaine can also be synthesized *de novo* from dietary choline in the mammalian liver and kidney *via* a two-step enzymatic process in which choline is first oxidized to betaine aldehyde by choline dehydrogenase. Then betaine aldehyde is again oxidized by betaine aldehyde dehydrogenase to produce betaine (Craig, 2004; Burg and Ferraris, 2008). The urinary excretion of betaine is minimal, and it is mainly eliminated from the body through metabolism (Schwab et al., 2006).

Although most mammalian cells are not exposed to extreme osmolalities, betaine is abundantly found in the liver and kidneys (Zhou et al., 2012; Kempson et al., 2014). Apart from *de novo* synthesis, betaine/γ-aminobutyric acid (GABA) transporter 1 (BGT1) facilitates the cellular uptake of dietary betaine from extracellular fluid (Zhou et al., 2012). BGT1 is primarily expressed in the liver, kidney, and brain (Zhou et al., 2012). Betaine has two essential functions in mammals (Lever and Slow, 2010). First, betaine serves as an osmoprotectant in the kidney. Since renal medullary cells, whose role is to concentrate urine, are constantly exposed to extremely high levels of sodium chloride and urea, the accumulation of betaine in renal medullary cells helps balance the hypertonicity in interstitial fluid (Burg and Ferraris, 2008; Kempson et al., 2014). The hypertonicity is reported to increase the number of BGT1 in the basolateral membrane (Schwab et al., 2006). Secondly, in the liver and kidney, betaine serves as a methyl group donor in betaine-homocysteine methyltransferase (BHMT, a zinc metalloenzyme) reaction in which betaine transfers its methyl group to homocysteine forming methionine (Millian and Garrow, 1998; Ueland, 2011). However, the function of betaine and BGT1 in the brain is poorly understood.

**FIGURE 3:**
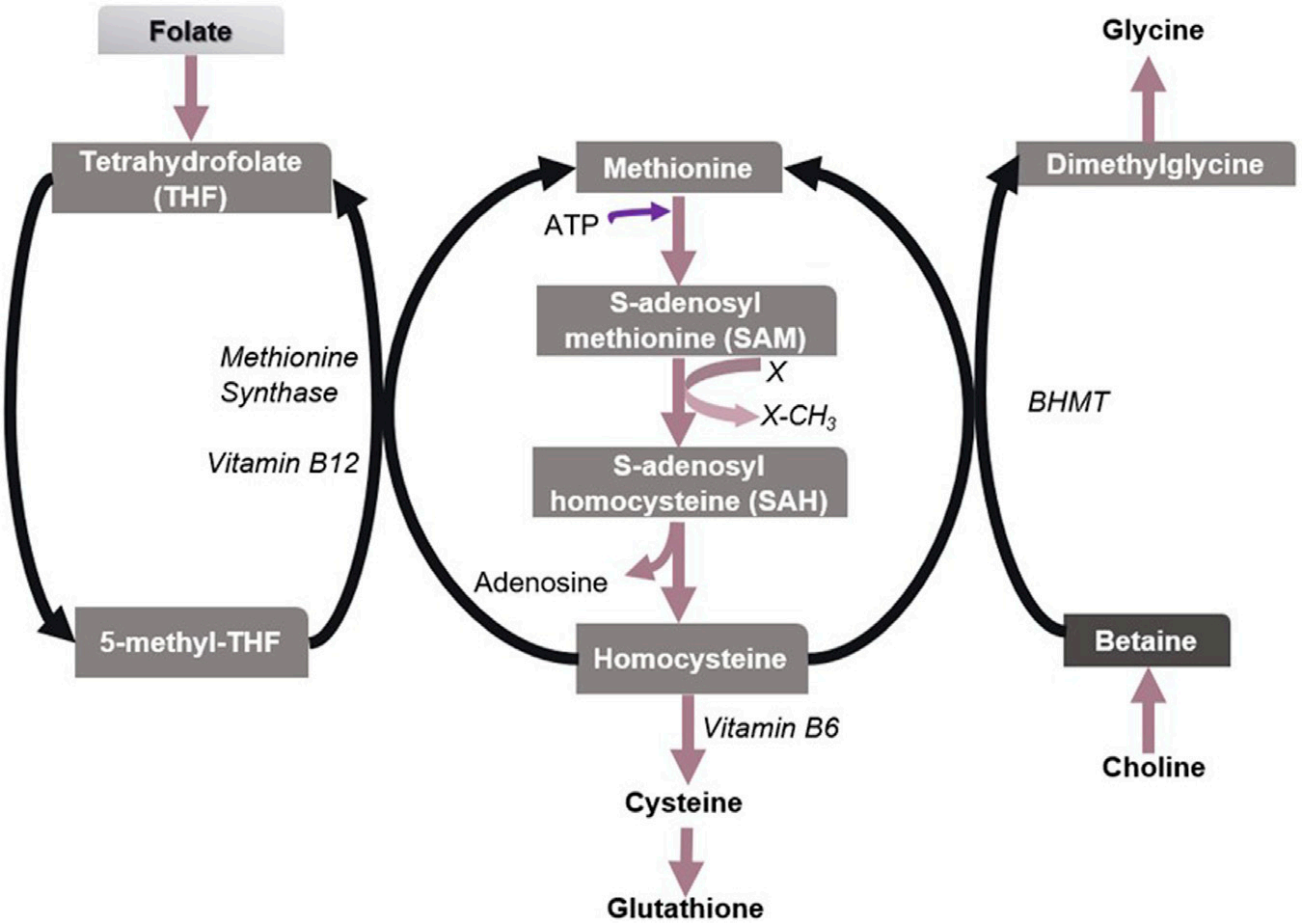
Metabolism of sulfur amino acids in the liver. Methionine/SAM, Folate/5-methyl-THF, and choline/betaine are important biological methyl group donors in methyl group transfer reactions. X denotes a methyl group acceptor. Betaine has been found to play a vital role in the methionine-homocysteine cycle and hence in maintaining the SAM/SAH ratio in the liver, especially when folate is insufficient.

Methionine is an essential proteinogenic amino acid and also the precursor of S-adenosylmethionine (SAM), the universal reactive methyl donor in biochemical reactions (Kharbanda et al., 2005). During the methyl group transfer, SAM becomes S-adenosylhomocysteine (SAH), which is then converted to homocysteine (Hcy). Hcy is a sulfur-containing non-protein amino acid, which is toxic to animals (Jakubowski, 2006). Hcy is detoxified by remethylating it to form methionine. This reaction recycles methionine and is generally catalyzed by methionine synthase that requires folate and vitamin B12. Alternatively, BHMT remethylates Hcy in which betaine acts as a methyl donor (Figure 3). However, the BHMT reaction is limited to the human liver and kidney since BHMT is expressed primarily in the hepatocytes and kidney cortex (Sunden et al., 1997). The deletion of Bhmt gene in mice resulted in an appreciable reduction in SAM/SAH ratio (i.e., the methylation potential) and a several-fold increase in hepatic and plasma total homocysteine (tHcy) concentrations (Teng et al., 2011). Hence betaine helps regulate the SAM/SAH ratio in the liver and plays a vital role in Hcy homeostasis.

## 3 Health implications of trimethylamine-N-oxide

**FIGURE 4:**
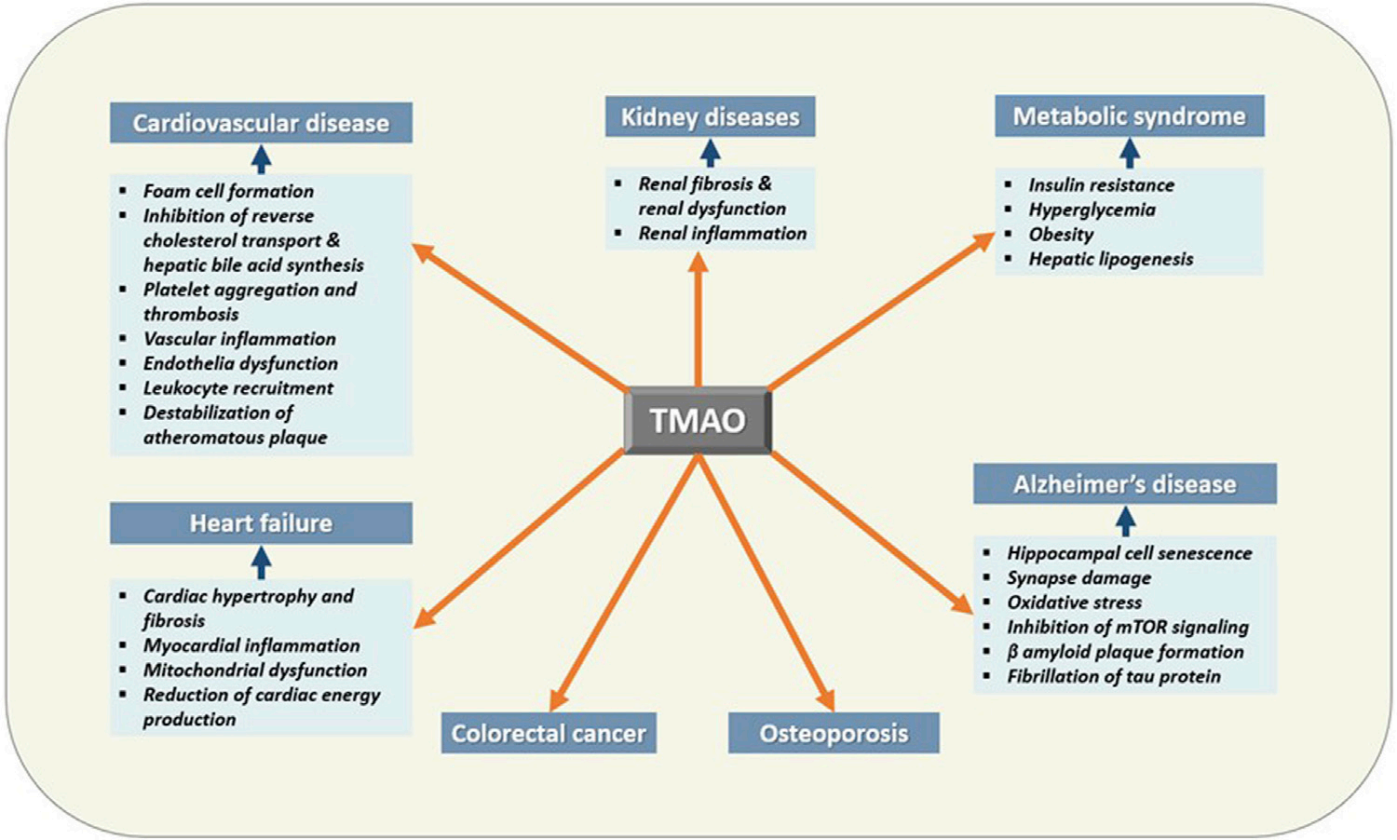
Effects of TMAO on human health and disease. TMAO is associated with the pathogenesis of several human diseases. Therefore, elevated serum TMAO can be considered a risk factor as well as can be used as a biomarker for these diseases.

TMAO has been extensively studied for its diverse roles in human health and disease other than being a universal osmoprotectant (Figure 4) (Table 1). The following sections summarize the recent findings of these studies.

**TABLE 1:** A summary of the health implications of TMAO with molecular mechanisms.

Disease	Effect of TMAO	Molecular mechanism	References
Atherosclerosis and cardiovascular disease	Enhances formation of foam cells	Upregulation of scavenger receptors (CD36, SR-A1) on macrophages	Wang et al. (2011), Geng et al. (2018)
	Inhibits reverse cholesterol transport	Unknown	Koeth et al. (2013)
	Inhibits hepatic bile acid synthesis	Downregulation of Cyp7a1 (cholesterol 7α-hydroxylase)	Ding et al. (2018), Geng et al. (2018)
	Promotes platelet aggregation and thrombosis	Enhancement of intracellular Ca^2+^ release in platelets	Zhu et al. (2016), Zhu et al. (2018)
	Promotes vascular inflammation, endothelial dysfunction, and enhances leukocyte recruitment	Elevates the levels of pro-inflammatory monocytes, Activation of the NLRP3 inflammasome, mitogen-activated protein kinase, and NF-kB	Seldin et al. (2016), Boini et al. (2017), Haghikia et al. (2018)
	Triggers instability of atheromatous plaques	Inhibition of macrophage M2 polarization and efferocytosis	Shi et al. (2021)
Heart failure	Cardiac hypertrophy and fibrosis	*via* Smad3 signaling	Li et al. (2019b), Li et al. (2019a)
		Activation of NLRP3 inflammasome	
	Myocardial inflammation	Activation of NLRP3 inflammasome	Chen et al. (2017b), Li et al. (2019b)
	Mitochondrial dysfunction and reduced cardiac energy production	Impairs pyruvate dehydrogenase activity and fatty acid beta-oxidation in mitochondria, promotes glycogen synthesis, and oxidative damage to proteins	Makrecka-Kuka et al. (2017), Savi et al. (2018)
Kidney diseases	Renal fibrosis and renal dysfunction (CKD)	Increases the expression of pro-fibrotic factor TGF-β1	Tang et al. (2015b), El-Deeb et al. (2019), Fang et al. (2021)
		Upregulation of Smad3 expression and promotes phosphorylation and activation of Smad3	
		Upregulation of kidney injury molecule-1	
	Renal inflammation	Activation of NLRP3 inflammasome	El-Deeb et al. (2019), Fang et al. (2021)
Metabolic syndrome	Insulin resistance	Blocks hepatic insulin signaling and triggers adipose tissue inflammation in mice	Gao et al. (2014)
	Hyperglycemia	Insulin resistance induces FMO3, which induces FoxO1	Miao et al. (2015)
	Insulin resistance→ increased hepatic FMO3 expression → increased TMAO synthesis	TMAO binds and activates PERK, which induces FoxO1	Chen et al. (2019)
	Obesity	FMO3, promotes obesity and is a negative regulator of beiging of white adipose tissue	Schugar (2017); Schugar et al. (2017)
	NAFLD	Modulate hepatic bile acid metabolism. Increase hepatic lipogenesis	Koeth et al., 2013, Tan et al. (2019)
Neurological disorders	Alzheimer’s disease is associated with elevated TMAO levels	Promotes hippocampal cell senescence, synapse damage, increases oxidative stress, and inhibits the mTOR signaling pathway	Li et al. (2018b), Vogt et al. (2018)
		Promotes β amyloid plaque formation	Yang et al. (1999); Scaramozzino et al. (2006)
		Promotes fibrillation of tau protein	
	Parkinson’s disease is associated with lower plasma TMAO levels	Unknown	Chung et al. (2021)
Osteoporosis	Decreases bone mineralization	Inhibits osteogenic differentiation and promotes adipogenic differentiation of bone marrow mesenchymal stem cells (BMSCs). Activates NF-κB signaling pathway and increases reactive oxygen species release and pro-inflammatory cytokine (IL-1β, IL-6, and TNF-α) production	Lin et al. (2020)
Cancer	Colorectal cancer is associated with elevated TMAO levels	Unknown	Bae et al. (2014), Liu et al. (2017)

### 3.1 Trimethylamine-N-oxide in cardiovascular diseases: A risk factor or a biomarker?

Cardiovascular diseases (CVDs) are the prime cause of morbidity and mortality worldwide (Roth et al., 2020). Mounting evidence suggests that increased circulating TMAO is a major actor in cardiovascular events in humans (Tang et al., 2013). A systematic review of 17 clinical studies involving over 25,000 subjects showed that increased systemic TMAO concentrations were correlated with higher cardiovascular events, and the relative risk of all-cause mortality was shown to increase by 7.6% per 10 μmol/L increments of plasma TMAO concentration (Schiattarella et al., 2017). In addition, Randrianarisoa et al. (2016) found that fasting serum TMAO levels correlated positively with carotid intima-media thickness (cIMT) independently of established CVD risk markers such as insulin resistance, fatty liver, and visceral obesity. cIMT is used as a predictor of cardiovascular events (Darabian et al., 2013). Moreover, based on the literature, Yao et al. (2020) suggested that TMAO levels of 5.1 μmol/L as a cut-off value for prognosis of many key unfavorable clinical events in patients with coronary heart disease.

In 2011, Hazen and colleagues discovered that TMAO is linked with the pathogenesis of CVDs in humans (Wang et al., 2011). They also demonstrated that dietary supplementation of apoE^−/−^ mice with choline, TMAO or betaine drives atherosclerosis by upregulating the scavenger receptors CD36 and SR-A1 on macrophages, leading to increased cholesterol accumulation and formation of foam cells (Geng et al., 2018). The TMAO-induced overexpression of macrophage CD36 receptor was later found to be mediated through MAPK/JNK pathway (Geng et al., 2018). In addition to choline, increased intake of dietary L-carnitine was also demonstrated to accelerate atherosclerosis in mice, which was observed to be linked with TMAO-induced inhibition of reverse cholesterol transport (Koeth et al., 2013).

The apoE^−/−^ mice fed with dietary TMAO for 8 weeks were found to have increased atheromatous plaques in the aorta and elevated serum lipids compared to the control group, which was demonstrated to be due to the inhibition of hepatic bile acid synthesis in mice possibly by downregulating Cyp7a1 (i.e., cholesterol 7α-hydroxylase, the key regulatory enzyme of hepatic bile acid biosynthesis) (Ding et al., 2018). Previously, Koeth et al. (2013) also noted that dietary TMAO supplementation significantly reduces the expression of Cyp7a1 and Cyp27a1 in mouse liver. Bile acid synthesis is the primary mechanism by which excess cholesterol is eliminated from the body (Chiang and Vlahcevic, 1996). Hence the inhibition of hepatic bile acid biosynthesis results in hypercholesterolemia. Apart from the atherosclerotic plaque size, plaque stability is also a risk factor for major adverse cardiovascular events (Shah, 2003). Shi et al. (2021) recently suggested that TMAO can activate an instability of carotid artery plaque in mouse models, possibly by inhibiting the M2 polarization and efferocytosis of macrophages.

Atherosclerosis is also identified as a chronic inflammatory disease in arteries (Raggi et al., 2018). Haghikia et al. (2018) reported that the increase of circulating proinflammatory monocytes in humans (intermediate CD14^++^CD16^+^ monocytes) and mice (Ly6C^high^ monocytes) are closely related to the elevated plasma TMAO concentrations. Intermediate CD14^++^CD16^+^monocytes have been found to be an independent predictor of cardiovascular events in humans (Rogacev et al., 2012). In addition, endothelial dysfunction and initiation of atherogenesis are known to trigger by activating the nuclear factor-κB (NF-κB) signaling pathway and NLRP3 (NOD-like receptor protein 3 or NLR family pyrin domain containing 3) inflammasome pathway in endothelial cells both *in vitro* and *in vivo* (Seldin et al., 2016; Chen et al., 2017a; Boini et al., 2017). Ma et al. (2017) observed that TMAO treatment promoted monocyte adhesion through upregulation of vascular cell adhesion molecule-1 (VCAM-1) but not the other adhesion molecules (i.e., intercellular adhesion molecule-1 (ICAM-1) and E-selectin) expression in human umbilical vein endothelial cells *in vitro*. However, TMAO was found to increase ICAM-1 expression together with tumor necrosis factor (TNF)-α and interleukin (IL)-6 in macrophages (Geng et al., 2018). In addition, TMAO was suggested to activate NF‐κB, likely through a G-protein coupled receptor Gβγ signaling pathway in endothelial cells and vascular smooth muscle cells, leading to enhanced expression of proinflammatory cytokines and leukocyte adhesion to endothelial cells *in vitro* (Seldin et al., 2016). Furthermore, TMAO-induced activation of the NLRP3 inflammasome, a proinflammatory protein complex, results in increased production of the proinflammatory cytokine IL-1β both *in vitro* and *in vivo*, which contributes to endothelial injury (Chen et al., 2017a; Boini et al., 2017). The activation of NLRP3 inflammasome was found to be associated with increased mitochondrial reactive oxygen species (ROS) generation due to the TMAO-induced downregulation of mitochondrial NAD^+^-dependent protein deacetylase sirtuin3 (Sirt3) and subsequent inhibition of manganese superoxide dismutase 2 (SOD2) activation (Rogacev et al., 2012; Fernando and Wijayasinghe, 2021). These results suggest that high plasma TMAO levels potentially contribute to chronic vascular inflammation and hence to increased incidence of cardiovascular events in patients.

It has also been observed that TMAO increases the risk of heart attack and stroke by modulating the susceptibility of thrombosis. In particular, TMAO was shown to trigger platelet activation and thrombosis *via* increasing the platelet responsiveness by enhancing stimulus (ADP, collagen, thrombin, or arachidonic acid)-dependent mobilization of intracellular Ca^2+^ stores in platelets (Zhu et al., 2016; Zhu et al., 2018). Inhibition of the gut microbial CutC/D, the TMA-generating enzyme reduced the plasma TMAO levels and rescued the platelet hyper responsiveness and thrombus formation in animal models. These studies revealed that inhibition of TMA or TMAO production is a prospective method to reduce the risk of thrombosis (Roberts et al., 2018).

As discussed above, elevated circulating TMAO levels correlate with a higher risk of adverse cardiovascular events. Hence, the inhibition of microbial TMA production provides a probable therapeutic approach for treating CVDs. In that regard, 3,3-dimethyl-1-butanol (DMB), a structural analog of choline has been found to inhibit microbial TMA lyase and reduced TMAO concentrations in mice fed with a choline or L-carnitine rich diet. DMB also inhibited foam cell formation and atherosclerotic lesion development in apoE^−/−^ mice (Wang et al., 2015).

However, some studies show no relationship between dietary choline, betaine, or carnitine with the incidence of CVDs, even though TMAO increases CVD mortality in some populations (Meyer and Shea, 2017; Samulak et al., 2019). It is also interesting to note that the people who consume large amounts of seafood (e.g., the Japanese population) have been reported to have high levels of TMAO in the urine but significantly low incidence of mortality due to heart diseases (Gatarek and Kaluzna-Czaplinska, 2021). Similarly, some animal studies have found protective effects of TMAO on CVDs. Moderate treatment of TMAO (∼6.7 mg/kg) was found to decrease diastolic dysfunction while increasing the TMAO level by four to five times in a hypertensive rat model (Huc et al., 2018). Moreover, in a recent review paper, Papandreou et al. (2020) presented convincing evidence that elevated TMAO in CVDs could result from the disease-related imbalance in gut microbiota but not the cause of CVDs (Huc et al., 2018). Therefore, it was suggested that TMAO is merely a biomarker of CVD progression.

In addition, TMA, the precursor of TMAO on human health and disease, has gained poor attention compared to TMAO. It was recently reported that CVD patients have two-fold higher plasma TMA concentrations than healthy individuals, indicating that TMA may also contribute to the pathogenesis of CVDs. Moreover, TMA but not TMAO was found to be toxic to human vascular smooth muscle cells and rat cardiomyocytes (Papandreou et al., 2020). Therefore, it was proposed that TMA but not TMAO is a marker of CVDs. In addition, age or disease (e.g., heart failure)-related alterations in the gut-blood barrier (i.e., leaky gut) resulted in increased plasma TMA levels in rats (Jaworska et al., 2019; Papandreou et al., 2020). Since the diversity of TMA-producing gut microbial community and their abundance varies from person to person and the effect of elevated TMA and TMAO on health appears to depend on the individual’s age and other underline pathologies. Further controlled studies are warranted to clarify above observed discrepancies. Furthermore, the validity of TMA/TMAO ratio as a better CVD biomarker needs to be investigated.

### 3.2 Trimethylamine-N-oxide is a predictor of mortality risk in heart failure

Heart failure (HF) increasingly contributes to global cardiovascular morbidity and mortality. Several studies have reported that TMAO affects heart functions and hence is identified as a risk marker for the prognosis of HF. In 2014, two research groups independently reported that circulating TMAO levels were significantly higher in patients with chronic heart failure than those without HF (median fasting TMAO value of 5.0 vs. 3.5 μM) (Tang et al., 2014; Trøseid et al., 2015). The raised plasma TMAO levels were found to be related to increased mortality risk in patients with stable/chronic HF independent of traditional risk factors, B-type natriuretic peptide (BNP) and renal function (eGFR) (Tang et al., 2014). With respect to TMAO, another study has noted that elevated choline and betaine also seem to contribute to the worsening of left ventricular diastolic dysfunction. However, only high concentrations of TMAO showed a prognostic value in chronic HF when adjusted for cardiorenal parameters (Tang et al., 2015a). Moreover, the increased TMAO levels, but not choline or betaine were found to be correlated with lower transplant-free survival during follow-up (Trøseid et al., 2015). Hence, the fasting TMAO levels were suggested to be useful in predicting 5-year mortality risk in HF patients. In addition, Suzuki et al. (2016) studied the association of TMAO with acute HF and found that circulating TMAO is an independent biomarker for predicting mortality and death/re-hospitalization due to HF within 1 year when renal function was excluded. When combined with NT-proBNP (N-terminal pro-B-type natriuretic peptide), the predictive value of death/HF was further strengthened.

Attempts have been made to study the effects of TMAO on cardiac muscle at the molecular level. TMAO was found to exacerbate myocardial hypertrophy and cardiac fibrosis in rodent models with transverse aortic constriction and also in mouse models of doxorubicin (DOX)-induced cardiac fibrosis (Organ et al., 2016; Li et al., 2019a; Li et al., 2019b). The TMAO treatment induced the hypertrophy of cultured cardiomyocytes and expression of hypertrophic markers such as atrial natriuretic peptide (ANP) and beta-myosin heavy chain (β-MHC) that was demonstrated to be mediated *via* TGF-β/Smad3 signaling that had been identified in the development of cardiac hypertrophy and fibrosis. These effects could be attenuated by lowering TMAO levels or pharmacological inhibition of Smad3 signaling (Li et al., 2019a). Subsequently, TMAO was demonstrated to stimulate cardiac fibrosis by activating the NLRP3 inflammasome in mouse primary cardiac fibroblast cultures (Li et al., 2019a). The NLRP3 inflammasome has been found to mediate collagen synthesis in cardiac fibroblasts (Zhang et al., 2018). In addition, TMAO was also found to increase the level of ROS and upregulate the expression of TLR4 in cultured cells (Li et al., 2019b). The increased oxidative stress and TLR4 also contribute to the activation of NLRP3 inflammasome and hence to cardiac fibrosis.

Moreover, TMAO was found to affect intracellular calcium handling and contractility of rat ventricular cardiomyocytes *in vitro* (Savi et al., 2018). Exposure to TMAO decreases pyruvate metabolism (*via* impaired substrate flux through pyruvate dehydrogenase), impairs β-oxidation, and affects energy metabolism in cardiac muscle fibers, leading to the development of HF (Makrecka-Kuka et al., 2017). Furthermore, TMAO-treated cardiomyocytes also revealed the build-up of glycogen and lipofuscin-like pigment, suggesting a mitochondrial dysfunction and lower ATP production that affects the contractile function of cardiomyocytes. TMAO-induced inhibition of pyruvate dehydrogenase results in impaired glycolysis. Therefore, glucose is diverted to glycogen synthesis. Savi et al. (2018) recently demonstrated that urolithin B and urolithin B-glucuronide could completely recover the TMAO-induced detrimental effects in cardiomyocytes. Urolithins are anti-oxidative and anti-inflammatory metabolites produced by gut microflora from dietary ellagitannins, which are polyphenolic compounds derived from ellagic acid and are rich in some fruits and nuts such as pomegranates, raspberries, walnuts, almonds, etc. Urolithin B has also been found to enhance skeletal muscle myotubes growth and differentiation *in vitro* and *in vivo* (Rodriguez et al., 2017).

### 3.3 Trimethylamine-N-oxide is a marker of renal impairment and a renal toxin

Since the kidney is the primary site of TMAO elimination from the body (Al-Waiz et al., 1987), impaired kidney function possibly results in elevation of TMAO levels in the blood. Indeed, TMAO has been suggested as a biomarker of renal impairment and elevated plasma TMAO levels (median TMAO 7.9 μmol/L) are found to be associated with chronic kidney diseases (CKD; eGFR <60 ml/min per 1.73 m^2^) and with poor prognosis in patients with CKD (Tang et al., 2015b). The mean serum TMAO concentration was reported to be markedly elevated in a cohort of patients who were new to hemodialysis (50 ± 32 μM [median 43 μM, interquartile range 28–67 µM]) compared to the control group with normal kidney function (1.41 ± 0.49 μM) (Kaysen et al., 2015). Another study also reported surprisingly high serum TMAO concentrations (median value 94.4 μM; interquartile range 54.8–133.0 μM) in dialysis-dependent patients, whereas healthy controls had a median TMAO concentration of 3.3 μM (interquartile range 3.1–6.0 μM) (Stubbs et al., 2016). Moreover, plasma TMAO was inversely associated with measured glomerular filtration rate (mGFR) since TMAO excretion was found to occur *via* glomerular filtration and the contribution of tubular secretion or reabsorption appeared to be insignificant in patients with CKD (Pelletier et al., 2019). Elevated TMAO significantly correlates with all-cause mortality in subjects with an estimated glomerular filtration rate (eGFR) of less than 90 ml/min per 1.73 m^2^ (Gruppen et al., 2017). The higher plasma TMAO level was found to be predictive of poor long-term survival in patients without CKD (eGFR ≥ 60 ml/min) irrespective of cystatin C levels implying that increase in TMAO levels may occur before the renal insufficiency (i.e., rise in cystatin C) (Tang et al., 2015b). The elevated TMAO levels could be reversed by hemodialysis (Bain et al., 2006) or kidney transplantation (Stubbs et al., 2016).

Besides, TMAO has also been identified as a renal toxin, which leads to the progression of kidney disease. The prolonged exposures to dietary TMAO or choline resulted in a significant increase in plasma cystatin C levels in mice, indicating renal dysfunction (Tang et al., 2015b). Moreover, feeding TMAO was found to further aggravate the development of diabetic kidney disease (DKD) in rats (Fang et al., 2021). In addition, the inhibition of TMA production with iodomethylcholine attenuated CKD in mice (Zhang et al., 2021). In animal models, the elevated TMAO levels were significantly related to tubulointerstitial fibrosis and collagen deposition in the kidney and TMAO was demonstrated to increase in kidney injury molecule-1, phosphorylation of Smad3 (Tang et al., 2015b). Smad3 is an important mediator of renal fibrosis (Meng et al., 2013). Fang et al. (2021) recently observed that oral administration of TMAO in DKD rats led to progressive tubulointerstitial fibrosis *via* upregulating transforming growth factor β (TGF-β), which is a central regulator of fibrogenesis and also exacerbated renal inflammation *via* activation of NLRP3 inflammasome.

### 3.4 Trimethylamine-N-oxide and metabolic syndrome

Emerging evidence suggests that TMAO contributes to the development of obesity and obesity-associated disorders. In a cross-sectional observational study, Barrea et al. (2018) found that circulating TMAO levels were positively related to body weight (body mass index, BMI), visceral adiposity index (VAI), and metabolic syndrome (MetS). VAI inversely correlates with insulin sensitivity (Amato et al., 2010). Hence, TMAO was proposed as a novel prospective indicator for the early prediction of MetS. The circulating levels of TMAO ≥8.74 µM were suggested as a cutoff for predicting the MetS (Barrea et al., 2018). Furthermore, the expression of FMO3 in subcutaneous white adipose tissue was found to be positively associated with obesity in mice and humans, and FMO3 was identified as a negative regulator of the beiging of white adipose tissue (Schugar et al., 2017). Furthermore, genetic deletion/knockdown of FMO3 stimulated beiging of white adipose tissue and conferred protection against obesity in mice (Schugar et al., 2017).

### 3.5 Trimethylamine-N-oxide in diabetes: Cause or effect?

Diabetes, in particular, type-2 diabetes mellitus (T2DM) is a major public health issue that causes significant morbidity and mortality globally. It is known that the insulin resistance (i.e., the insensitivity of cells, in particular, hepatocytes, skeletal muscle cells, and adipocytes to insulin, the primary hormone in glucose homeostasis in the fed state) predisposes to T2DM. An early study identified that gut microbiota-derived methylamines or decreased choline bioavailability perform an appreciable role in the progression of insulin resistance and non-alcoholic fatty liver disease (NAFLD) in high-fat diet-fed mice (Dumas et al., 2006). Subsequently, TMAO is identified as a significant modulator in the progression of T2DM (Kim et al., 2015), and higher fasting plasma TMAO levels have been observed in patients with T2DM (Shan et al., 2017; Tang et al., 2017). Zhuang et al. (2019) conducted a systematic analysis that included pooled data from 12 clinical studies with over 15,000 participants around the world and reported that circulating TMAO concentrations were positively correlated with a high risk of diabetes mellitus, and the odds ratio for T2DM prevalence was reported to increase more than 50% per 5 μmol/L rise of plasma TMAO.

Several animal studies have also suggested that TMAO may be a risk factor for insulin resistance and diabetes. Dietary TMAO was found to aggravate the impaired glucose tolerance, interfere with insulin signaling in the liver, and trigger adipose tissue inflammation in mice fed a high‐fat diet (Gao et al., 2014). Moreover, the diabetic db/db mice exhibited significantly higher TMAO concentrations, increased body weight, and insulin resistance compared to non-diabetic db/L mice, demonstrating that diabetes and BMI are positively related to circulating TMAO levels (Dambrova et al., 2016). Miao et al. (2015) suggested that FMO3, the TMAO-producing enzyme is under the control of insulin signaling, and also FMO3 is required for FoxO1 (Forkhead box O1) transcription factor expression. FoxO1 is found to play a vital role in regulating glucose production in the liver (Gross et al., 2009). Insulin suppresses hepatic FMO3 expression while glucagon elevates expression of FMO3 at both mRNA and protein levels. FMO3 levels were found to be increased in obese or insulin resistance in animals and humans (Miao et al., 2015). The increase of circulating TMAO concentrations seems to mediate primarily by hepatic insulin resistance *via* induction of FMO3. Therefore, the measures that improve hepatic insulin sensitivity could lower the plasma TMAO levels, which would ameliorate the cardiometabolic risk associated with high TMAO.

Moreover, Chen et al. (2019) recently reported that hepatic endoplasmic reticulum (ER) stress kinase PERK (protein kinase R-like endoplasmic reticulum kinase) is a receptor for TMAO. At pathological concentrations (50 μM), TMAO was found to bind and activate PERK both *in vitro* and *in vivo*, which in turn, induced FoxO1 and thereby upregulates the gluconeogenic genes *G6pc* (glucose-6-phosphatases), *pck1* (cytosolic phosphoenolpyruvate carboxykinase) in the liver, leading to hyperglycemia. Knockdown of FMO3 was demonstrated to suppress FoxO1 and improve glucose tolerance in mice (Miao et al., 2015).

### 3.6 Trimethylamine-N-oxide in non-alcoholic fatty liver disease

A case-control and a cross-sectional study revealed that the presence and extremity of non-alcoholic fatty liver disease (NAFLD) were significantly correlated with higher circulating TMAO levels in Chinese adults (Chen et al., 2016). Moreover, Barrea et al. (2018) identified that circulating levels of TMAO were positively associated with fatty liver index (FLI), a surrogate marker of NAFLD. The circulating levels of TMAO ≥8.02 µM suggested the presence of NAFLD-FLI. Thus, TMAO was suggested as an initial biomarker of NAFLD-FLI (Zhang et al., 2021). Furthermore, Flores-Guerrero et al. (2021) reported that plasma TMAO concentrations were positively and independently correlated with baseline FLI (≥60), and increased TMAO was correlated with a high risk of all-cause mortality in subjects with NAFLD.

Another study reported that increased circulating TMAO levels were notably related to non-alcoholic steatohepatitis (NASH) in Mexican obese patients in the presence of T2DM and the circulating secondary bile acids were found to be associated both with elevated TMAO levels and NASH (Leon-Mimila et al., 2021). Tan et al. (2019) suggested that the serum TMAO levels were positively correlated with the serum total bile acids concentrations, particularly the percentage of taurocholic acid (a farnesoid X receptor (FXR) antagonist bile acid), resulting in increased expression of bile acid synthesis enzyme CYP7A1 (cholesterol 7α-hydroxylase) in NAFLD patients. TMAO administration in HFD-fed mice elevated hepatic lipogenesis and increased bile acid synthesis. Similarly, TMAO treatment enhanced lipogenesis in palmitic acid-treated HepG2 cells. These findings demonstrated that TMAO exacerbates hepatic steatosis by altering bile acid metabolism and by suppressing FXR signaling in the liver (Tan et al., 2019). However, it has previously been demonstrated that TMAO supplementation markedly downregulated the expression of critical enzymes in bile acid biosynthesis (CYP7A1 and CYP27A1) and hence reduced the size of the bile acid pool (Koeth et al., 2013). The disparity between the findings of the two studies could be due to the dissimilarity in animal models and experimental setup. Yet, further studies are required to better reflect the mechanistic view by which TMAO influences the development of NAFLD.

### 3.7 Trimethylamine-N-oxide in neurodegenerative diseases

The recent evidence demonstrates a link between TMAO and several other gut microbial-derived metabolites with neurological diseases (Xu and Wang, 2016). Vernetti et al. (2017) using a human microphysiological system (i.e., organs-on-a-chip) model, demonstrated that TMAO could penetrate the blood-brain barrier. In fact, TMAO has been found in cerebrospinal fluid (CSF) of individuals with neurological disorders at concentrations 0.11–6.43 µmol/L (Del Rio et al., 2017). However, this study did not determine whether there is a correlation between plasma and CSF TMAO levels in the study cohort. A subsequent study showed that TMAO concentrations in CSF were notably higher in patients with cognitive impairments and with Alzheimer’s disease in contrast to cognitively unimpaired individuals, and elevated CSF TMAO was suggested as a biomarker of AD pathology (Vogt et al., 2018). Plasma TMAO concentrations were observed to be increased with age in humans and mice (Li et al., 2018b; Gao et al., 2019a). Li et al. (2018b) reported that TMAO could instigate brain aging and age-related cognitive decline in mice. They found that TMAO promotes cell senescence in the CA3 region, damages the ultrastructure of synapse in the CA1 region, and increases oxidative stress in the hippocampus of mice. TMAO has also been found to promote plaque formation by stabilizing and adjusting the assembly of the amyloid-β (Aβ) peptide (Yang et al., 1999) and fibrillation of tau protein by inducing the secondary structure formation at the C terminus *in vitro* (Scaramozzino et al., 2006). These changes may lead to neurodegeneration and AD. Moreover, the inhibition of TMA lyase with DMB was found to alleviate neuroinflammation, reduce Aβ peptide formation (due to decreased β secretase activity), and hence ameliorated the cognitive deterioration in mice (Gao et al., 2019a). In contrast, lower plasma TMAO levels (<6.92 μmol/L) were found to be associated with Parkinson’s disease (PD) and suggested as a biomarker in early PD and also suggest the notion that a higher level of TMAO might slow down the progression of PD (Chung et al., 2021). Moreover, decreased plasma TMAO, choline, and butyrobetaine levels were found in amyotrophic lateral sclerosis (ALS) patients compared to healthy controls, suggesting a disturbance in the metabolism and/or absorption of these metabolites in the gut in ALS patients (Chen et al., 2020). However, additional studies are required to validate the function of elevated systemic TMAO concentrations in the progression of different age-associated neurological disorders.

### 3.8 Trimethylamine-N-oxide and cancer

The intestinal bacteria contribute to the oncogenesis and progression of several human cancers *via* inflammatory and metabolic mechanisms (Zitvogel et al., 2015). Hence manipulation of gut microbiota has been suggested for cancer therapy. Several recent reports have shown that the microbial metabolite TMAO influences colorectal carcinogenesis. In an early study, Bae et al. (2014) observed that higher plasma TMAO concentrations were related to an increased risk of colorectal cancer (CRC) among postmenopausal women, especially in women who had low plasma vitamin B12 levels. Another study found a significantly higher serum TMAO levels in CRC patients (range 5.6–15.8 μmol/L) compared to the healthy controls (range 2.0–4.8 μmol/L) and serum TMAO was proposed as a novel prognostic marker in patients with colorectal cancer (Liu et al., 2017). It appears that TMAO involves in a number of genetic pathways implicated in colon cancer (Xu et al., 2015a). Yet, mechanistic details about how TMAO causes CRC is unknown.

## 4 Health implications of betaine

**FIGURE 5:**
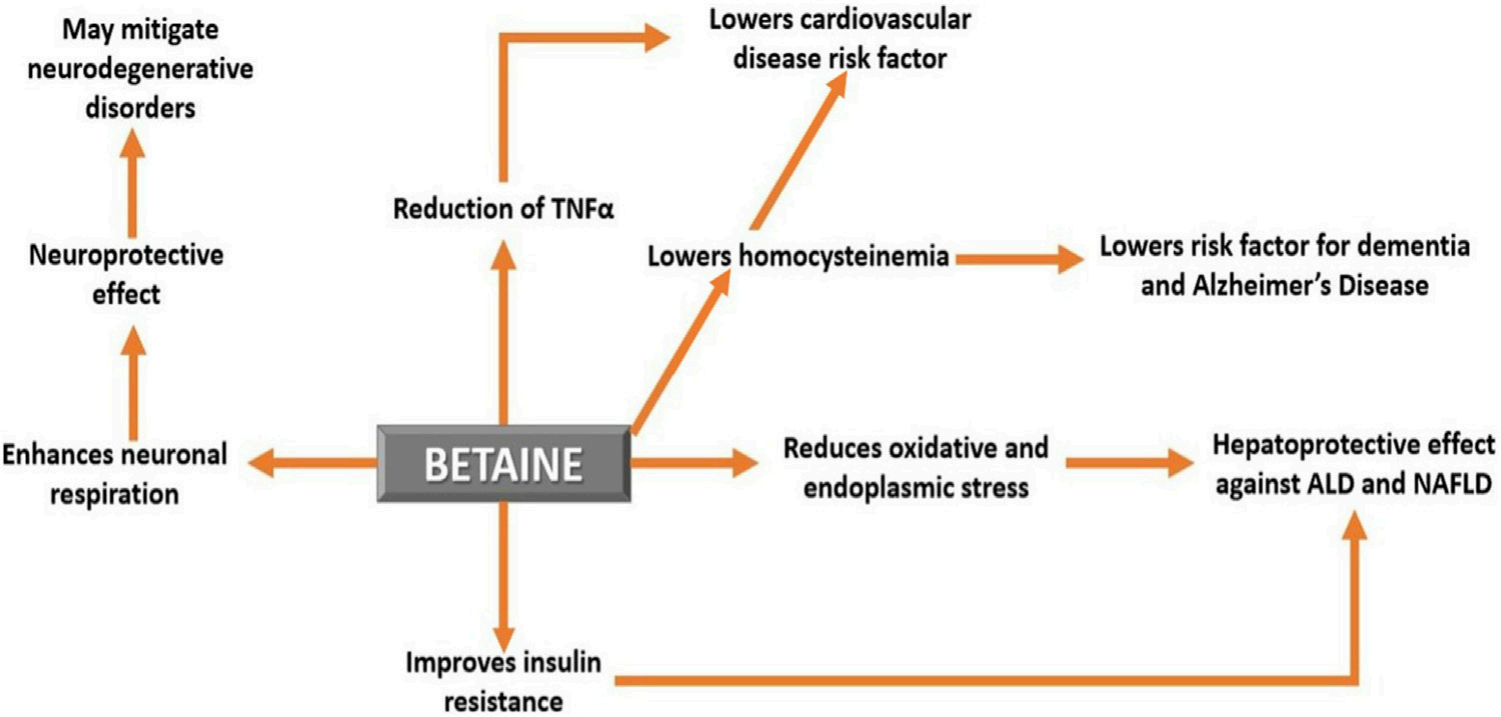
The effect of betaine on human health and disease. Betaine demonstrates a number of beneficial effects and therefore can be used as a potential therapy for several human diseases.

Compared to TMAO, betaine has a number of beneficial effects on human health and disease (Figure 5) (Table 2). In healthy humans, the plasma betaine concentration ranges from 20 to 70 μmol/L (Lever et al., 1994; Craig, 2004).

**TABLE 2:** A summary of the health implications of betaine with molecular mechanisms.

Disease	Effect of betaine	Molecular mechanism	References
Homocystinuria	Lowers plasma tHcy levels	Promotion of methionine-homocysteine cycle	Craig (2004)
Cardiovascular disease	Lowers plasma tHcy levels	Promotion of methionine-homocysteine cycle	Schwab et al. (2006), Olthof et al. (2016)
	Reduction of inflammation	Lowers the C-reactive protein, IL-6, and TNF-α	Detopoulou et al. (2008), Lv et al. (2009)
Liver disease (Alcoholic and non-alcoholic fatty liver disease)	Suppresses hepatic lipid synthesis	suppression of DGAT1, DGAT2, SREBP-1c, SREBP-2, fatty acid synthase, and HMG-CoA reductase and upregulation of PGC-1α in the liver	Yang et al. (2017)
	Enhances fatty acid oxidation	Increase of the expression of CPT1, PPARα, FGF21, and AMPK in the liver	Li et al. (2015b)
	Promotes VLDL synthesis and release	Increase of hepatic SAM/SAH ratio and promotes one-carbon metabolism and synthesis of phosphatidylcholine	Barak et al. (1997), Deminice et al. (2015)
		Enhances BHMT expression and promotion of hepatic PC synthesis	Li et al. (2015b)
	Alleviates hyperhomocysteinemia	Promotion of methionine-homocysteine cycle	Kenyon et al. (1998), Stickel et al. (2000)
	Reduction of oxidative stress	Increase in cellular reduced glutathione levels	Jung et al. (2013), Zakhari (2013), Kim et al. (2017)
	Reduces ER stress	Attenuation of GRP78, CHOP, and JNK activation	Kaplowitz and Ji (2006), Wang et al. (2010)
	Inhibition of inflammatory response	Suppression of NLRP3 inflammasome activation	Kim et al. (2017)
		Inhibition of IL-1β production and release	Xia et al. (2018), Zhao et al. (2018)
		Induction of IL-10 and decreasing TNF and IL-6 expression	Veskovic et al. (2019)
	Improves adipocyte functions	Increase in mitochondrial biogenesis	Du et al. (2018)
		Corrects aberrant adipokine production	Wang et al. (2010)
	Improves insulin resistance	Activation of IRS1, PKB/Akt, and AMPK	Kathirvel et al. (2010); Du et al. (2018)
	Modulate epigenetic modifications	Increase of SAM/SAH ratio and restore methylation capacity; Downregulation of fatty acid synthase and upregulation of fatty acid oxidation (ACOX, PPARα, AMPK, FGF10, ATGL)	Wang et al. (2014); Chen et al. (2021)

		Increase in mitochondrial content and activity	Zhang et al. (2019)
	Inhibits apoptosis	Reduction of Bax and induction of Bcl-2	Veskovic et al. (2019)
		Activation of Akt/mTOR signaling	Veskovic et al. (2019)
	Activates autophagy	Increases the expression of beclin 1, Atg4, and Atg5	Veskovic et al. (2019)
Neurological disorders	Enhances neuronal mitochondrial respiration	Modulation of histone H3 trimethylation on Lys 4 (H3K4me3) in neurons	Singhal et al. (2020)
	Promotes oligodendrocyte maturation	Modulation of DNA methylation and increases the expression of oligodendrocyte maturation genes SOX10 and NKX-2.2	Sternbach et al. (2021)
	Prevents oxidative stress	Enhancement of glutathione peroxidase activity	Alirezaei et al. (2011)
	Mitigates hyperhomocysteinemia in Alzheimer’s disease	Attenuation of tau hyperphosphorylation and Aβ production	Chai et al. (2013)

### 4.1 Betaine in hyperhomocysteinemia

The elevated plasma total homocysteine (i.e., hyperhomocysteinemia) is a known risk factor for CVDs (Refsum et al., 1998) and is also associated with some neurological disorders (Ansari et al., 2014) and cancer (Hasan et al., 2019). The fasting plasma tHcy levels are known to be negatively associated with folate and vitamin B12 intake. Cho et al. (2006) found that dietary betaine and choline (the precursor of betaine) intake was inversely related to plasma tHcy levels independent of folate and other B vitamins (B6 and B12) intake. This association was prominent when folate intake was inadequate, indicating that the function of betaine in methyl group metabolism is vital in folate deficiency. On average, a daily intake of 12 g (9–15 g) is reported to be safe and a 6 g/day dose of betaine can lower the elevated circulating Hcy levels in the hereditary disorder called homocystinuria, which is caused by inborn errors in Hcy metabolism (Craig, 2004). Therefore, in addition to folate and vitamin B12, betaine helps to lower the Hcy levels in the blood and betaine insufficiency may be considered a possible cause for hyperhomocysteinemia.

### 4.2 Betaine in cardiovascular diseases

Hyperhomocysteinemia is a risk factor for CVDs (Refsum et al., 1998). Betaine is used clinically to lower plasma Hcy levels in homocystinuria (Truitt et al., 2021). It has been shown that oral betaine intake at 6 g/day decreased the fasting plasma homocysteine concentrations significantly in healthy humans (Olthof et al., 2003; Schwab et al., 2006; Olthof et al., 2016), and betaine could reduce the increase in Hcy after methionine level has been increased to 50% (Olthof and Verhoef, 2005). Methionine-loading test is used as an indicator of the propensity of the individual to heart disease (Garlick, 2006). Moreover, a low dose of betaine, similar to dietary betaine (∼0.5–2 g/day) intake found to lower the fasting plasma Hcy by 16%. Thus, it was concluded that a betaine-rich diet might reduce CVD risk (Olthof et al., 2003). In addition, the higher intake of choline and betaine were correlated with a reduction of serum inflammatory markers, such as C-reactive protein (CRP) and TNF-α in healthy adults (Detopoulou et al., 2008), indicating a decrease of inflammation that may contribute to the reduction of CVD mortality. Betaine insufficiency was observed to be correlated with a higher risk of secondary acute myocardial infarction and heart failure in acute coronary syndrome patients (Lever et al., 2012). Betaine supplementation was shown to decrease atherosclerotic lesion area in apoE deficient mice and that was found to be linked with the betaine-induced reduction of TNF-α expression in the aorta (Lv et al., 2009). Oral betaine treatment for 7 days in a rat model of cholestasis-associated renal injury was found to decrease oxidative stress markers, improve mitochondrial indices, and alleviate histopathological changes in the kidney (Ommati et al., 2021).

Nevertheless, a significant increase in total and LDL-cholesterol levels in the blood have been reported with betaine treatment in overweight individuals with metabolic syndrome and obese individuals but not in healthy subjects (Schwab et al., 2002; Turck et al., 2017). Zawieja et al. (2021) recently reviewed six randomized controlled trials that estimated the effect of betaine supplementation (at least 4 g/day) in adults for a maximum of 24 weeks on blood lipids and found that total cholesterol was increased moderately, but there was no significant effect on plasma LDL and HDL cholesterol or TG levels. However, the molecular mechanism by which betaine increases blood cholesterol concentrations is yet to be explored. Another systematic review of six prospective cohort studies with a total of 1,84,010 participants with 18,076 incident CVD events has found no positive relationship between dietary choline or betaine intake and CVD incidence (Meyer and Shea, 2017). Even though it appears that betaine adversely affects blood cholesterol, which can be outweighed by the promising valuable effects of betaine treatment on CVDs.

### 4.3 Betaine in liver disease

Alcoholic liver disease (ALD) and non-alcoholic fatty liver disease (NAFLD) are the most prevalent liver diseases worldwide and currently have no effective treatment. Although the pathogenesis of these liver diseases is complex, both show a similar spectrum of liver pathology that ranges from steatosis (i.e., the buildup of fat) to steatohepatitis (i.e., the inflammatory state), cirrhosis (i.e., the fibrosis/scarring) and hepatocellular carcinoma (Toshikuni et al., 2014). Bhmt deleted mice were observed to have significantly high hepatic triglycerides and later developed hepatic tumors (Teng et al., 2011), indicating that reduced BHMT activity may increase the susceptibility to fatty liver and hepatocellular carcinoma. A number of studies have suggested that betaine is hepatoprotective and could ameliorate liver injury in animal models of ALD and NAFLD through a number of molecular mechanisms, including enhancing hepatic triglyceride export and oxidization, reduction of oxidative and ER stresses, prevention of inflammatory response, improvement of insulin resistance, promotion of transmethylation reactions and modulation of epigenetic modification.

#### 4.3.1 Alcoholic liver disease

The liver is the primary site of alcohol metabolism. Ethanol metabolism produces toxic molecules such as acetaldehyde and ROS (Cederbaum, 2012). Therefore, excessive and chronic alcohol consumption can alter liver biochemistry leading to liver disease. Some of these changes include lowering of NAD^+^/NADH ratio, changes in redox state, increased fat synthesis, hepatocellular damage, and other associated malfunctioning of metabolic activities (Cederbaum, 2012).

Betaine supplementation was demonstrated to alleviate the alcohol-induced fatty liver in rats by improving hepatic lipid metabolism (i.e., decreasing the synthesis and increasing the catabolism) *via* suppression of diacylglycerol acyltransferases: DGAT1, DGAT2, sterol regulatory element binding proteins: SREBP-1c, SREBP-2, fatty acid synthase, and HMG-CoA reductase and overexpression of peroxisome proliferator-activated receptor λ coactivator (PGC)-1α in the liver and preventing the decline of adiponectin concentrations in serum and adipose tissue (Yang et al., 2017).

Chronic alcohol intake is known to alter the methionine cycle in the liver. Acetaldehyde, which results from the oxidation of ethanol in the liver, inhibits methionine synthase activity, causing a lowering of hepatic SAM concentration and concurrent increase of SAH level and hyperhomocysteinemia (Kenyon et al., 1998; Stickel et al., 2000). In addition, excessive ROS formation can lower the methionine availability to form SAM as Hcy is used to form glutathione (Zakhari, 2013). Betaine can restore hepatic SAM levels.

The ethanol-induced biochemical changes (i.e., elevated serum triglycerides, liver transaminases, TNF-α levels, intrahepatic lipid accumulation, and reduced antioxidant capacity) in rats were effectively reversed by betaine supplementation. This was claimed to be associated with the enhancement of antioxidant defense in the liver (Jung et al., 2013). Moreover, betaine prevented the liver from ethanol-induced steatosis in rats by increasing the hepatic SAM levels (Barak et al., 1997).

Hyperhomocysteinemia is known to induce ER stress, which can activate sterol regulatory element-binding proteins (SREBPs) and upregulate enzymes in lipid synthesis, contributing to triglycerides and cholesterol accumulation and also induces apoptosis (Kaplowitz and Ji, 2006; Kaplowitz et al., 2007). Betaine supplementation in alcohol-fed mice was found to decrease hyperhomocysteinemia, hepatic lipids, and ER stress response (Ji and Kaplowitz, 2003).

#### 4.3.2 Non-alcoholic liver disease

In an early pilot study, the non-alcoholic steatohepatitis (NASH) patients who received betaine anhydrous oral solution at a dose of 20 g/day for 1 year resulted in a significant improvement in liver biochemistry and histology. Moreover, betaine was well tolerated and safe (Abdelmalek et al., 2001). It has been reported in another case-control study that the severity of the liver disease is associated with betaine insufficiency, as betaine levels were observed to be significantly decreased in NASH compared to NAFL (Sookoian et al., 2017). Betaine treatment was demonstrated to lessen non-alcoholic hepatic steatosis in animals in a dose-dependent manner (Wang et al., 2014).

Phosphatidylcholine (PC or lecithin) is a constituent in lipoproteins, the lipid transport particles. Impairment of hepatic PC synthesis has been linked to hepatic lipid accumulation since it affects very-low-density lipoprotein (VLDL) production and secretion. Two pathways are involved in the synthesis of PC: 1) CDP-choline pathway and 2) from phosphatidylethanolamine (PE). PE is converted to PC *via* a series of methylation reactions that use SAM as the methyl group donor. (van der Veen et al., 2017). Therefore, low SAM levels affect the PC synthesis in the hepatocytes, resulting in the accumulation of lipids in the liver. Betaine enhanced the activity and expression of BHMT, as well as elevated the PC and VLDL levels, thus improving hepatic lipid export. (Xu et al., 2015b). Betaine improved methylation capacity by increasing SAM/SAH ratio and normalized the gene expression of key enzymes accountable for one-carbon metabolism (e.g., BHMT, GNMT, and MGAT) associated with the regulation of fat metabolism in the liver (Brosnan et al., 2015).

Anomaly in gene expression in hepatocytes due to aberrant DNA methylation leads to the disorder of NAFLD. Wang et al. (2014) suggested that treatment of betaine ameliorates NAFLD in the mice fed with a high-fat diet possibly by restoring the methylation capacity by increasing the SAM/SAH ratio and thereby reversing the abnormal expression of the genes involved in lipid metabolism, such as FAS (fatty acid synthase), ACOX (acyl-CoA oxidase), PPARα (peroxisome proliferator-activated receptor alpha) in the liver. In addition, betaine significantly upregulated the protein expression of AMPK (AMP-activated protein kinase), FGF10 (fibroblast growth factor 10), and ATGL (adipose triglyceride lipase) and suppressed hepatic lipid accumulation in mice, suggesting that betaine prevents NAFLD *via* FGF10/AMPK signaling pathway (Chen et al., 2021).

Betaine supplementation was observed to decrease histopathological alterations and serum markers of hepatotoxicity in animal models of acute and chronic non-alcoholic liver injury. Those effects were claimed to be attributed to the betaine-induced mitigation of oxidative stress and protection of hepatocyte mitochondria by betaine (Heidari et al., 2018). Betaine seems to improve hepatic fat metabolism also by promoting mitochondrial content and activity as these effects were shown to mediate by betaine-induced RNA methylation in cultured HepG2 cells (Zhang et al., 2019).

Obesity is a paramount risk factor for NAFLD. The concentration of plasma betaine was observed to be inversely proportional to the percentage of fat in Chinese adults (Chen et al., 2015). A meta-analysis of six randomized controlled trials with 195 participants showed that betaine supplementation could significantly reduce body fat (Gao et al., 2019b). In an animal study, dietary betaine supplementation was found to limit obesity *via* inhibiting new white adipose tissue formation, enhancing lipid metabolism through increasing mitochondrial biogenesis and also improved insulin resistance (Du et al., 2018).

NAFLD is associated with insulin resistance. Oral betaine administration was shown to decrease fasting glucose concentrations, improve insulin-resistance and thereby alleviate NAFLD in high-fat diet-fed insulin resistance NAFLD mouse models (Kathirvel et al., 2010; Wang et al., 2010). Betaine treatment increased the phosphorylation and activation of insulin receptor substrate 1 (IRS1), protein kinase B (PKB/Akt), and also promoted AMPK in HepG2 cells (Kathirvel et al., 2010). Betaine enhanced fatty acids oxidation as evidenced by the increase in the expression of carnitine palmitoyltransferase 1 (CPT1, a key regulatory enzyme in fatty acid β-oxidation) and other regulatory proteins: PPARα, FGF21, and AMPK in the liver (Xu et al., 2015b). In addition, betaine improved adipose tissue function in high-fat diet-fed mice as shown by the corrected aberrant adipokine (adiponectin, resistin, and leptin) production, relieved ER stress, and enhanced insulin sensitivity (Wang et al., 2010). These effects may in part contribute to reverse the insulin-resistant in hepatocytes and alleviate NAFLD. The dietary betaine (or choline) intake was not associated with the risk of type 2 diabetes in humans (Dibaba et al., 2020).

The long-term inflammatory response in the liver could result in liver fibrosis, leading to cirrhosis. Betaine was shown to suppress NLRP3 inflammasome activation and IL-1β production in the liver of diabetic mice by inhibiting insulin-induced activation of the FoxO1 transcription factor, leading to the activation of thioredoxin and hence suppression of ROS production in hepatocytes (Kim et al., 2017). ROS triggers inflammation by activation of NLRP3 inflammasome (Mittal et al., 2014). Betaine shows anti-inflammatory effects both by inhibiting proinflammatory IL-1β production and IL-1β release (Xia et al., 2018; Zhao et al., 2018). Hence, betaine therapy may be beneficial not only in liver disease but also in other health conditions which are linked with inflammation, such as obesity, heart diseases, and Alzheimer’s disease (Mathieu et al., 2010; Fernando and Wijayasinghe, 2021).

Veskovic et al. (2019) recently showed that the oral betaine treatment not only restored the methionine-Hcy cycle, but also reduced hepatosteatosis, oxidative stress (*via* increasing glutathione levels and antioxidant enzyme activities), inflammation (*via* induction of anti-inflammatory IL-10 and decreasing proinflammatory TNF and IL-6 expression), and apoptosis (*via* reduction of proapoptotic mediator Bax and induction of anti-apoptotic mediator Bcl-2), but also found to activate autophagy (*via* increase the expression of beclin 1, Atg4 and Atg5, the autophagy activators) and prosurvival Akt/mTOR signaling in the liver of methionine and choline deficient diet (MCD)-induced NAFLD mice.

### 4.4 Betaine in neurodegenerative diseases

McDonough and colleagues recently reported that BHMT is expressed in the brain and betaine enhances neuronal respiration and promotes oligodendrocyte maturation by modulating histone and DNA methylation. Therefore, it was suggested that betaine is neuroprotective, and dietary betaine supplementation may mitigate neurodegenerative disorders (Singhal et al., 2020; Sternbach et al., 2021). Since betaine regulates the concentration of SAM in the cell, betaine can influence the methylation status and hence modulates the expression of several genes in cellular metabolism (Figueroa-Soto and Valenzuela-Soto, 2018). Betaine depletion and aberrant DNA and histone methylation in the brain have been identified to be associated with multiple sclerosis (Singhal et al., 2015). Hyperhomocysteinemia is a risk factor for dementia and Alzheimer’s disease (Seshadri et al., 2002). The restoration of the plasma homocysteine level with folate and vitamin B12 combined treatment has been reported to attenuate Alzheimer’s pathology (i.e., Aβ accumulation, tau hyperphosphorylation) and memory impairment (Chai et al., 2013). The betaine supplementation was also demonstrated to ameliorate the Hcy-induced memory deficit and attenuate the tau hyperphosphorylation and Aβ production in a hyperhomocysteinemic rat model (Chai et al., 2013). In addition, oral administration of betaine was shown to prevent hyperhomocysteinemia and oxidative stress in the cerebellum of ethanol-fed rats (Alirezaei et al., 2011) and as well as the in the brain of levodopa and benserazide (agents used in the therapy of Parkinson’s disease) treated rats (Alirezaei et al., 2015), showing the beneficial effects of betaine in the neurological disorders.

## 5 Conclusion and future perspectives

Besides being compatible osmolytes that stabilize cellular proteins, allowing them to survive in adverse environmental conditions, TMAO and betaine play diverse roles in human health and disease. The present review summarized the up-to-date knowledge of the multifaceted functions of these two key osmolytes in humans.

TMAO is synthesized in the liver from TMA produced by the intestinal bacteria in dietary choline and L-carnitine catabolism. Therefore, the gut microbiome contributes significantly to the concentration of TMAO in the body. TMAO is a metabolic waste product of choline and L-carnitine and is excreted in the urine. TMAO is toxic to the kidney and has been shown to have severe implications in the progression of many diseases such as cardiovascular diseases, heart failure, kidney disease, metabolic syndrome, neurological diseases, and some cancers. Therefore, TMAO has been considered a risk factor for cardiovascular diseases, heart failure, and marker of renal impairment. However, research suggests that not only TMAO but also TMA is a marker in cardiovascular pathologies. The contradicting factors support future research where TMA/TMAO can be used as a potential biomarker for numerous human diseases. Since most of these findings are based on animal studies, the role of TMA and TMAO in humans needs to be investigated.

Betaine can be obtained either from diet or synthesized from dietary choline in the liver and kidney. In the kidney, betaine helps stabilize cellular proteins from urea and it acts as a methyl group donor in the liver. In contrast to TMAO, betaine exhibits beneficial effects on human health, it could be used as a promising preventive therapy for liver disease, cardiovascular disease, neurodegenerative disease, etc. Betaine helps regulate plasma tHcy levels *via* BHMT reaction, which is significant in folate insufficiency. In addition, the elevated tHcy may be a sign of the inadequacy of betaine. It is also worth investigating whether betaine deficiency is associated with the pathogenesis or with the severity of the above diseases. Betaine is antioxidant and anti-inflammatory and hence has the potential to ameliorate the diseases associated with oxidative stress and inflammation. Even though preclinical data are compelling, the clinical studies on betaine are sparse. Given the benefits of betaine therapy in animal models, controlled clinical trials need to be conducted to evaluate the effectiveness of betaine therapy in various human diseases as betaine is safe and low-cost.

## Glossary

ACOX: Acyl-CoA oxidase
AD: Alzheimer’s disease
ALD: Alcoholic liver disease
ALS: Amyotrophic lateral sclerosis
AMPK: AMP-activated protein kinase
ANP: Atrial natriuretic peptide
ATGL: Adipose triglyceride lipase
Aβ: Amyloid Beta
BHMT: Betaine-homocysteine methyltransferase
BMI: Body mass index
BNP: B-type natriuretic peptide
cIMT: carotid intima-media thickness
CKD: Chronic kidney diseases
CntA/B: Carnitine oxygenase/reductase
CPT1: Carnitine palmitoyltransferase 1
CRC: Colorectal cancer
CRP: C-reactive protein
CutC: Choline TMA-lyase
CVDs: Cardiovascular diseases
Cyp7a1: Cholesterol 7α-hydroxylase
DKD: Diabetic kidney disease
DM: Diabetes mellitus
DMB: 3,3-dimethyl-1-butanol
DOX: Doxorubicin
ER: Endoplasmic reticulum
FAS: Fatty acid synthase
FGF: Fibroblast growth factor
FLI: Fatty liver index
FMO3: Flavin-containing monooxygenase 3
FoxO1: Forkhead box O1
FXR: farnesoid X receptor
G6pc: Glucose-6-phosphatases
GABA: Betaine/γ-aminobutyric acid
GrdH: Glycine betaine reductase
Hcy: Homocysteine
HF: Heart failure
ICAM-1: Intercellular adhesion molecule-1
IL: Interleukin
IRS1: Insulin receptor substrate 1
MCD: Methionine and choline deficient diet
MetS: Metabolic syndrome
mGFR: measured Glomerular filtration rate
NAFLD: Non-alcoholic fatty liver disease
NASH: Nonalcoholic steatohepatitis
NF-κB: Nuclear factor-κB
NLRP3: NOD-like receptor protein 3
NT-proBNP: N-terminal pro-B-type natriuretic peptide
PC: Phosphatidylcholine
Pck1: Phosphoenolpyruvate carboxykinase
PD: Parkinson’s disease
PE: Phosphatidylethanolamine
PERK: Protein kinase R-like endoplasmic reticulum kinase
PKB: Protein kinase B
PPARα: Peroxisome proliferator-activated receptor α
ROS: Reactive oxygen species
SAH: S-adenosylhomocysteine
SAM: S-adenosylmethionine
SOD2: Superoxide dismutase 2
SREBPs: Sterol regulatory element-binding proteins
T2DM: Type-2 diabetes mellitus
TG: Triglyceride
TGF-β: Transforming growth factor β
tHcy: total homocysteine
TMA: Trimethylamine
TMAO: Trimethylamine N-oxide
TNF: Tumor necrosis factor
VAI: Visceral adiposity index
VCAM-1: Vascular cell adhesion molecule-1
VLDL: Very low-density lipoprotein
β-MHC: Beta-myosin heavy chain
γBB: γ-butyrobetaine
